# Better Regulation of End-Of-Life Care: A Call For A Holistic Approach

**DOI:** 10.1007/s11673-022-10213-8

**Published:** 2022-10-17

**Authors:** Ben P. White, Lindy Willmott, Eliana Close

**Affiliations:** Australian Centre for Health Law Research, Faculty of Business and Law, Queensland University of Technology, GPO Box 2434, Brisbane, Queensland 4001 Australia

**Keywords:** End-of-life care, Medical legislation, Government regulation, Ethical issues

## Abstract

Existing regulation of end-of-life care is flawed. Problems include poorly-designed laws, policies, ethical codes, training, and funding programs, which often are neither effective nor helpful in guiding decision-making. This leads to adverse outcomes for patients, families, health professionals, and the health system as a whole. A key factor contributing to the harms of current regulation is a siloed approach to regulating end-of-life care. Existing approaches to regulation, and research into how that regulation could be improved, have tended to focus on a single regulatory instrument (e.g., just law or just ethical codes). As a result, there has been a failure to capture holistically the various forces that guide end-of-life care. This article proposes a response to address this, identifying “regulatory space” theory as a candidate to provide the much-needed holistic insight into improving regulation of end-of-life care. The article concludes with practical implications of this approach for regulators and researchers.

## Introduction

Regulation plays an important part in the effective functioning of a health system (Vincent et al. 2020). Through instruments such as law, policies, guidelines, ethical codes, training, and funding programs (Vincent et al. 2020), regulation aims to ensure the safety and quality of healthcare provided to the community. In our field of interest–end-of-life care–effective regulation is critical. Appropriate decision-making about life-sustaining treatment, system access to palliative care, and the safe regulation of voluntary assisted dying (where lawful) all affect the quality of end-of-life care and all depend on effective regulation. Despite this, existing regulation in this field across countries is flawed. This leads to adverse outcomes for patients, families, health professionals, and the health system. As such, end-of-life care–a field well recognized as a troublesome area for healthcare improvement (Wolf, Berlinger, and Jennings 2015; Curtis et al. 2012)–provides an ideal, albeit challenging, case study to reflect on how best to design and evaluate health regulation.

We contend that end-of-life regulation must be designed and evaluated taking a holistic approach. Existing approaches to regulation of end-of-life care and how it could be improved have tended to focus on a single regulatory instrument (e.g., just law or just policy). As a result, work to date has failed to capture holistically the various forces that guide end-of-life care. This article begins by identifying why effective regulation of end-of-life care is important before outlining the harms of current regulatory approaches for patients, families, health professionals, and the health system. It considers the factors that contribute to those harms, with a particular focus on the absence of a holistic perspective on regulation of end-of-life care. The article then proposes an analytical approach for how to address this, identifying “regulatory space” theory as a candidate to provide the much-needed holistic insight into improving regulation of end-of-life care. It concludes with practical observations for both regulators and researchers about the implications of the proposed holistic approach.

A final introductory point to note is that what constitutes “regulation” is contested (Black 2002; Koop and Lodge 2017). Historically, regulation has often been used to refer only to formal coercive regulatory instruments issued by the State, such as law (Black 2002). However, increasingly regulation is viewed as a broader concept and indeed, such an approach underpins the call outlined below to widen how regulation of end-of-life care is understood. For our purposes, we adopt Julia Black’s widely-used definition: “regulation is the sustained and focused attempt to alter the behaviour of others according to defined standards or purposes with the intention of producing a broadly identified outcome or outcomes, which may involve mechanisms of standard-setting, information-gathering and behaviour-modification” (Black 2002, 26). Such a definition recognizes that regulation can occur through a range of instruments such as law, policies, guidelines, ethical codes, training, and funding programs.^1^ This definition of regulation also acknowledges the role of entities (regulatory actors) who create these regulatory instruments or tools or oversee compliance with them, and recognizes that these entities may or may not be part of the State (Black 2002).

To give a practical illustration, our conception of regulation means that when a doctor is deciding whether to provide treatment they consider to be non-beneficial, we are interested in whether law is relevant to their decision or is trumped by hospital policy. Or perhaps neither is known by the doctor, let alone influential in decision-making. Instead, it may be that professional guidelines from a medical college or society for managing the patient’s illness, or ethical guidelines from a health quality commission about communication and managing conflict at the end of life, are most persuasive. Analysis of regulation of end-of-life care has not been approached in this holistic way to date, but we consider this is important as explained further below.

## Effective Regulation of End-Of-Life Care is Important

The nature of end-of-life care and the decisions it entails demonstrates why effective regulation, with its standard setting and oversight, is important. First, end-of-life care involves decisions of a serious and grave nature. Often these decisions result in death, for example, through withholding or withdrawing life-sustaining treatment, alleviating symptoms through potentially life-shortening doses of palliative medication, or intentionally causing death (voluntary assisted dying or euthanasia) (Löfmark et al. 2008).^2^

Secondly, these end-of-life decisions occur frequently, at least in countries with advanced health systems (van der Heide et al. 2003). For example, national surveys undertaken in relation to medical end-of-life decision-making have estimated that medical decisions precede around 56 per cent of all deaths in the United Kingdom (Seale 2009) and 65 per cent of all deaths in Australia (Kuhse et al. 1997). That amounts to approximately 340,000 and 110,000 deaths each year in these countries respectively (Office for National Statistics 2019; Australian Bureau of Statistics 2019).^3^ It is also anticipated that these numbers will grow in line with the sharp increases predicted for the number of deaths over the next twenty years (Swerissen and Duckett 2014).

Thirdly, not only will the frequency of these decisions increase with an ageing population, so too will their *complexity*. Increasing prevalence of dementia will lead to more end-of-life decisions involving the uncertainty of deciding on behalf of another. Advances in medicine will facilitate keeping people alive for longer but with questions about the utility of their poorer health states and the significant cost of their care and treatment (Callahan 2011). Societal shifts about expectations of control over medical decisions by members of the Baby Boomer generation (born between 1946 and 1964) and wider moves toward patient consumerism will continue to alter existing paradigms of decision-making (Callahan 2011). Further, international trends to legalize voluntary assisted dying will see increasing numbers of this more controversial type of end-of-life decision (White and Willmott 2018).

## Current Regulation of End-Of-Life Care is Flawed

While effective regulation can improve end-of-life care (Carlson et al. 2008), poor or ineffective regulation can lead to sub-optimal care (Stewart 2011; Hoffman and Tarzian 2005). Existing literature suggests that current regulation across a range of countries is flawed and not operating as intended, leading to adverse outcomes for patients, families, health professionals and health systems.

For *patients and families*, poor end-of-life regulation, including lack of knowledge or misperceptions about it, can lead to patients receiving unnecessary and potentially harmful futile or non-beneficial treatment (Willmott et al. 2016; White et al. 2020b).^4^ It can also contribute to inadequate participation by patients and substitute decision-makers in decisions about medical treatment (Visser, Deliens and Houttekier 2014) or a failure to offer patients appropriate life-saving treatment (Office of the Public Advocate (Queensland) 2016). Complex and bureaucratic regulation can even preclude patient access to some end-of-life care with evidence of this occurring in the prescriptive and highly-regulated voluntary assisted dying system in Victoria, Australia (Willmott et al. 2021﻿; White et al. 2021b). Further, uncertainty of health professionals about legal protections available to them has been linked to patients receiving inadequate pain and symptom management (Stewart 2011; Berlinger, Jennings, and Wolf 2013). Health professionals lack sufficient knowledge that there is strong legal, ethical, and policy support for palliative care intended to relieve pain even if it may hasten death by a short period (Willmott et al. 2020).

Poor regulation can also lead to adverse outcomes for *health professionals*. Unclear law, policy, or guidelines can introduce legal, ethical, and other risks or harms into decision-making for health professionals (Hawryluck, Oczkowski, and Handelman 2016; Downar, Warner, and Sibbald 2016; Department of Health (NSW) 2010; Close et al. 2021). One example is health professionals who are “accidental non-compliers”: those who intend to comply with the law (an advance directive in this study) but the law’s complexity or counter-intuitive position means they that they inadvertently do not (White et al. 2017b). Another example is moral distress caused by providing or witnessing futile or non-beneficial treatment in part because health professionals are unclear about legal, ethical, and policy support to avoid such treatment (Mobley et al. 2007; Dzeng et al. 2016). Others argue that such distress can directly arise from law or policy that they consider unjust or misguided. In contrast to earlier examples, the law or policy may be clear and known, but create conflict for health professionals by requiring them to choose between regulatory compliance and fulfilling their perceived ethical and clinical duties to patients (Hawryluck, Oczkowski, and Handelman 2016; Downar, Warner, and Sibbald 2016). Health professionals have also reported burdens of navigating complex and bureaucratic voluntary assisted dying regulation (Willmott et al. 2021; White et al. 2021b; Pesut et al. 2020).

Poor end-of-life regulation also adversely affects *health systems* as a whole. Where law or policy is not known or is perceived not to support clinical decisions, this can lead to waste through defensive medicine or overtreatment (Willmott et al. 2016). An Australian study about futile treatment (which cited regulatory concerns as one of a number of reasons such treatment was provided) estimated 12 per cent of studied hospital admissions included care not benefitting patients, resulting in a projected national cost of A$153 million (Carter et al. 2017). U.S. (Huynh et al. 2013) and Canadian (Schouela et al. 2021) studies have also quantified the cost of non-beneficial or futile treatment within particular health centres, with the Canadian study specifically reporting on costs for patients where disputes meant legal adjudication was required or considered. Inadequate regulation such as the lack of justifiable, transparent decision-making processes and effective dispute resolution mechanisms can also be costly and create inefficiencies and conflict with patients, families, and treating clinicians (Department of Health ﻿(NSW) 2010; Sibbald, Chidwick, and Hawryluck 2014; Wilkinson and Savulescu 2018). Finally, “perverse financial incentives” can distort healthcare practice reducing the health system’s ability to deliver high quality end-of-life care (Institute of Medicine 2015, 15).

We are not suggesting that all, or even most, challenges in delivering end-of-life care are regulatory ones. However, the foregoing literature shows the many ways in which poor end-of-life regulation leads to adverse outcomes for patients, families, health professionals, and health systems. Inadequate regulation (whether through its design or how it is implemented) adversely affects the safety and quality of end-of-life care, and this is particularly so in light of the inclusive definition of regulation we adopt. This points to the need to improve this regulation, part of which includes examining the reasons why it is flawed.

## Factors Contributing to Flawed Regulation of End-Of-Life Care

Regulation of end-of-life care is flawed in three linked ways. The first is that poor design of some individual regulatory instruments results in a failure to achieve their intended outcomes. At a macro level, the Institute of Medicine has argued that funding programs in the United States fail to incentivize the care that is needed at the end of life (Institute of Medicine 2015). A more micro example is a recent critique of voluntary assisted dying laws in Victoria, Australia, which demonstrated that significant parts of that legislation are directly inconsistent with its own stated policy goals (White et al. 2020a).

Regulation may also struggle to achieve its purpose because it is so complex and contradictory that it cannot give clear guidance to patients, substitute decision-makers, and health professionals. To illustrate, adult guardianship and medical treatment legislation in New South Wales, Australia, creates multiple possible substitute decision-makers (White et al. 2011):

- whose powers are activated by different tests for decision-making incapacity;
- whose legal powers to make key decisions vary with some decisions permitted and others not depending on which substitute decision-maker is empowered to decide; and
- who must apply different legislative criteria when making decisions, again depending on which substitute decision-maker is deciding.

A second problem is that specific regulatory instruments, even if well-designed, may lack the normative force needed to guide behaviour as intended. This point does not evaluate the regulation’s quality but rather focuses on its limitations in influencing intended targets. For example, law is not well known by doctors (White et al. 2014; White et al. 2021a), nurses (Willmott et al. 2020; White et al. 2021a), and patients and families (Tilse et al. 2019). Law is also often not followed with, for example, studies showing some doctors reporting not following advance directives they know to be binding (White et al.﻿ 2017a; Moore et al. 2019; Hardin and Yusufaly 2004). Policies, guidelines, and ethical codes also have limited influence on end-of-life care (Goodridge 2010; Hawryluck 2006). Medical training in end-of-life care does not teach this area well and lacks traction in guiding clinical practice (Parker et al. 2015).

While there has been a body of work on these two problems, very little attention has been directed to the third: that current regulation lacks a holistic approach to guiding end-of-life care. This siloed approach to regulation is reflected in much of the above literature, which focuses only on a single instrument of regulation (e.g., just law or just policy). This piecemeal approach is ineffective because it leads to fragmented and potentially conflicting guidance and misses the critical overall perspective of how law, policies, ethical codes, training, funding programs, and other regulatory forces interact to guide end-of-life decision-making. In other words, regulation of end-of-life care to date has been seen as a siloed undertaking where law *or* policy *or* ethical codes are regarded as both the problem to address and the source of the solution, instead of considering how a wide range of regulatory instruments currently function together, and could do so more effectively.^5^

The need for a holistic approach to end-of-life regulation can be surmised from at least some existing empirical literature. For example, one study based on surveying doctors about compliance with a legally binding advance directive suggested that the normative force of law appears to be trumped by ethical or clinical considerations (White et al. 2017a). Another study drawing on qualitative interviews with doctors about following advance directives reached a similar view (Moore et al. 2019). Although not explicitly holistic studies, they looked beyond the single regulatory instrument being studied to consider how other factors may also shape behaviour. They found there is intersection, and indeed conflict, between different regulatory forces, supporting the need to examine holistically how end-of-life care is regulated. The need for a holistic approach can also been seen in normative work such as *The Hastings Center Guidelines for Decisions on Life-Sustaining Treatment and Care Near the End of Life* (Berlinger, Jennings, and Wolf 2013). These ethics guidelines support engagement with ethical reasoning when making end-of-life decisions but also integrate other regulatory forces such as law, policy, clinical guidelines, and training.

One advantage of such a holistic approach is that it also accounts for the other two problems mentioned above: poor regulatory design and a failure to exert intended normative force. Indeed, a holistic approach *requires* seeing the intersections between these three linked problems. It would enable, for example, understanding how a policy which is poorly-designed (e.g., it does not properly reflect the relevant clinical context) is not likely to be respected or followed by doctors. Further, part of what may make the policy poorly-designed is an assumption that the relevant policy was the sole regulatory instrument that would guide behaviour, hence failing to account for the impact of other instruments such as law or ethical codes.

## A Proposed Holistic Approach to Regulating End-Of-Life Care

Having called for a holistic approach to regulating end-of-life care, what might it look like? There are a range of regulatory theory models that adopt a more holistic approach (Morgan and Yeung 2007) and common to them are three key points, which emerge from the preceding discussion. First, a holistic approach to regulation requires stepping beyond just law, which historically has been the major focus of regulation (Black 2002). Other regulatory instruments such as policy, guidelines, ethical codes, training, and funding programs must also be examined.^6^ Secondly, a holistic approach to regulation means that it is not the sole province of the State. If regulation is conceptualized as the “attempt to alter the behaviour of others according to defined standards or purposes” (Black 2002, 26) and this can be done through instruments other than law, then non-State actors can (and do) regulate. Thirdly, the interaction of these regulatory actors and instruments is crucial; they do not operate in isolation from each other.

One holistic approach we propose as apposite for designing and evaluating regulation of end-of-life care, with its multiplicity of intersecting regulatory actors and instruments, is regulatory space theory. First proposed by Hancher and Moran in the context of business regulation (Hancher and Moran 1989), this theory conceptualizes a “regulatory space” that is occupied by a series of regulatory actors and their corresponding instruments of regulation, all competing to influence behaviour within that defined arena. This theory has been used recently in the health setting to examine regulation of patient safety (Oikonomou et al. 2019), biobanks (Kaye et al. 2012), precision medicine (Nicol et al. 2016), and research ethics (Burris 2008; Laurie 2017).

The first stage in this approach is to map the relevant regulatory actors and instruments. Examples of possible actors and their common instruments in an end-of-life “regulatory space” are outlined in Table 1. We stress the illustrative nature of these examples and note particularly that the actors within a specific regulatory space will depend on location and context (e.g., the state or country and the nature and organization of its health system). We also note the possible intersection between regulatory instruments. To illustrate, funding programs may be established by legislation or policy, and training for health professionals may include information about their legal duties.

**Table 1:** Examples of Possible Regulatory Actors and Instruments in an End-of-Life Regulatory Space

**Actors**	**Instruments**					
	***Law***	***Policies***	***Guidelines***	***Ethical Codes***	***Training***	***Funding Programs***
**Parliaments and Courts** e.g., federal, state/provincial and/or local (depending on a country’s system of government)	✓ Legislation and court decisions					✓
**Governments and their departments** e.g., federal, state/provincial and/or local governments and departments (depending on a country’s system of government and how their healthcare system is administered)	✓ Generally delegated/ subordinate legislation	✓	✓		✓	✓
**Statutory agencies and bodies** e.g., local health authorities; safety and quality commissions; clinical excellence bodies; health and medical licensing boards; therapeutic goods and pharmaceutical regulators; clinical ethics advisory bodies; directors of public prosecutions; coroners	✓ Some via delegated/ subordinate legislation or enforcement of law	✓	✓	✓	✓	✓
**Health service organizations (public and private)** e.g., hospitals, residential aged and/or disability care providers, hospices and community care providers		✓	✓	✓	✓	✓
**Non-government organizations** e.g., health and medical colleges and societies; health and medical associations and unions; patient advocacy groups; private health insurers		✓	✓	✓	✓	✓

This mapping then involves identifying the influence of actors and instruments to guide the behaviour of others, and how these influences compete to determine what conduct is prohibited, permitted, or encouraged. This exercise produces a comprehensive map of the regulatory space that addresses: What issues are regulated; by which (overlapping) actors and instruments; how is influence exerted and by whom; and how is competing influence from instruments and actors resolved to guide behaviour? (Kaye et al. 2012). Such an approach can, for example, address questions such as: What regulatory instruments most influence doctors’ decisions about end-of-life care? How is conflicting guidance navigated, e.g., when law proscribes treatment but policy or training would permit it to be given? This approach should also involve careful scrutiny for actors or instruments that appear to be missing: this could show a lack of influence or reveal hidden power.

In addition to regulatory space theory enabling a comprehensive understanding of current frameworks, it can also be used to support regulatory reform (Scott 2001). This could involve evaluating how existing actors and instruments of regulation could function together better to promote high quality end-of-life care. It may also point to a need to create a new regulator or instruments (Black 2003), or to remove them if they are not improving the quality of end-of-life care (Oikonomou et al. 2019). A “clean slate” approach could be taken: while learning lessons from the past, existing structures are put aside to consider anew how best to regulate end-of-life care.

## Some Practical Observations About a Holistic Approach to Regulating End-Of-Life Care

### For Regulatory Actors

This section shifts from the theoretical to make some practical observations and we begin with two such observations aimed at current or potential regulatory actors in light of the proposed holistic approach. The first is that those producing a regulatory instrument must anticipate its operation within a wider regulatory space with other regulatory actors and instruments competing to guide behaviour. To illustrate, if a medical college or society is producing a policy about advance care planning, they must be conscious of existing law, policy, guidelines, ethical codes, and training which already aim to guide the behaviour of doctors, other health professionals and patients and families in this area.

This consideration of how regulatory instruments might work together includes having regard to the usual nature and function of each instrument type. For example, law often grants power to individuals to make decisions about treatment but provides limited guidance about operationalization of these decisions. Policies are often needed to translate abstract legal rules into a clinical context and guidelines may provide concrete processes for doctors and other health professionals to follow. Training may contribute by providing the skills required to provide high quality end-of-life care, such as the ability to have difficult conversations. In short, individual regulatory efforts should strive to see the wider picture so that consistent guidance is provided, using the strengths of different regulatory instruments to guide behaviour to desirable outcomes.

A second practical observation for regulatory actors, and particularly the State, is that instead of incremental reform of individual regulatory instruments, there may be windows to reform end-of-life regulation in a broader or more holistic way. An illustration of this is the implementation of Victoria’s voluntary assisted dying legislation. An eighteen-month implementation period prior to the law beginning enabled the Victorian Government to develop the necessary regulatory infrastructure to support the law, including: an oversight board, guidelines for clinicians, policy guidance for health services, training for medical practitioners, and information and education for the community (Department of Health & Human Services (Victoria) 2021). Although the Victorian Government was understandably focused on aspects of this new law controlled by the State (non-State actors were not fully integrated into this exercise), this more holistic approach to designing regulation would resonate with the model we are proposing.

### For Researchers

Practical observations about holistic approaches can also be made to inform the approach of researchers studying end-of-life regulation. First, it remains appropriate for research about a single regulatory instrument or actor to continue. Such focused research endeavours provide an important knowledge base that facilitates wider holistic research. However, an analysis of a single regulatory instrument or actor should at least aim to situate that instrument or actor within the context of the wider regulatory space. At the very least, this would include acknowledging it is one of a number of factors that guides behaviour. There may also be scope to consider its interaction or relationship with those other factors. Concrete conclusions may not be possible due to study design but “peering over the disciplinary fence” to at least consider relationships with other regulatory instruments or actors would support development of this wider holistic approach. Examples were given above of both empirical and normative work that has done this.

A second observation is that researchers should strive to undertake holistic regulatory studies of the sort described above, for example, drawing on regulatory space theory or other holistic approaches. Such work is obviously a more ambitious undertaking and would require greater resourcing than the narrower single instrument studies and may also need interdisciplinary perspectives. However, for the reasons outlined above, current regulation of end-of-life care is flawed and we argue that holistic research is needed to address this.

### Limitations

A final set of practical observations is to briefly acknowledge limitations of the proposed holistic approach. One is that such an approach provides very little guidance as to the desirable normative content of end-of-life regulation. Apart from some higher-level direction such as the need to comply with basic principles of good governance (e.g., regulation should be clear and consistent), this branch of regulatory theory focuses on optimizing the effectiveness of regulation achieving its intended policy outcomes. It does not determine what those outcomes should be, and that normative content must be found elsewhere.

A second observation is that a holistic approach, with its recognition of the regulatory role of non-State actors, acknowledges challenges for the coordination and legitimacy of regulation. Setting and implementing an overall regulatory direction may be difficult when competing goals are being pursued by multiple regulatory actors with different interests, and when compliance depends on acceptance of authority to set and enforce standards. We recognise this challenge but still consider that regulation is likely to be better if this challenge is acknowledged and regulation designed with this reality in mind.

A third observation is to acknowledge that even holistic evaluations of end-of-life regulation would need to stop at some point. To illustrate, end-of-life care is shaped by the wider health system, which in turn is shaped by the wider political system and social context. A complete examination of these increasingly wider settings is unlikely to be feasible or even possible so practical and conceptual boundaries will still need to be drawn for the sorts of holistic examinations of end-of-life regulation we are proposing.

## Conclusion

This article calls for a new holistic approach to designing and evaluating regulation of end-of-life care. Citing harms of current regulation, in part caused by a siloed or isolated approach to considering discrete regulatory instruments and actors, the article proposes that a wider holistic perspective is required. Regulatory space theory was suggested as one appropriate approach and it was explained how it could address current deficits. The article concludes by making practical observations about the implications of this holistic approach both for regulatory actors and researchers.

We should be clear that we do not consider that this approach will be a panacea for addressing suboptimal regulation of end-of-life care. Regulation is always imperfect and end-of-life care is a challenging and complex area of clinical practice to govern. Nevertheless, we consider a holistic approach will help advance our understanding of regulation and its design and evaluation, which will in turn improve the quality of end-of-life care. We also consider that efforts to improve regulation of end-of-life care, an area historically resistant to improvement (Wolf, Berlinger, and Jennings 2015; Curtis et al. 2012), may provide insight for regulating other areas of clinical decision-making and help advance the broader field of health regulation.

## Footnotes

1 We note that the nature of these regulatory instruments and their scope are debated. It is not necessary for the purposes of this article to engage with these debates; it is sufficient to note that a breadth of regulatory instruments exist and this article argues that they need to be considered in an integrated way when designing and evaluating regulation of end-of-life care.

2 We note that decisions to intentionally cause death occur not only in jurisdictions where such practices are lawful but also where they are not (Löfmark et al. 2008). As will be discussed, regulation is not always effective in guiding behaviour towards its desired outcomes.

3 The authors make these estimates based on the number of total deaths most recently reported by the jurisdictions’ national statistics offices multiplied by the percentage of deaths that are preceded by medical decisions. For the United Kingdom, this is the Office for National Statistics. For Australia, this is the Australian Bureau of Statistics.

4 We note too, as an aside, that terms such as “futile” or “non-beneficial” treatment are contested (White et al. 2020b).

5 We note, for example, that law may be underpinned by a set of policy justifications or ethical beliefs. However, such references to policy and ethics are different from the regulatory instruments being considered here, namely a policy document or an ethical code. It is this latter sense of these concepts in which our argument about siloing of regulatory instruments is made. We thank an anonymous referee for highlighting this distinction.

6 Interestingly, the historical focus on law in the regulatory literature as the primary instrument of regulation has tended not to be an issue in the health and medical literature on end-of-life care as it has considered more often the regulatory impact of instruments such as policies, guidelines, ethical codes, and training. Indeed, until quite recently, there has been relatively limited focus on the role of law in end-of-life care.

## Declarations

This research did not require ethical approval.
